# Progressively Disrupted Intrinsic Functional Connectivity of Basolateral Amygdala in Very Early Alzheimer’s Disease

**DOI:** 10.3389/fneur.2016.00132

**Published:** 2016-09-19

**Authors:** Marion Ortner, Lorenzo Pasquini, Martina Barat, Panagiotis Alexopoulos, Timo Grimmer, Stefan Förster, Janine Diehl-Schmid, Alexander Kurz, Hans Förstl, Claus Zimmer, Afra Wohlschläger, Christian Sorg, Henning Peters

**Affiliations:** ^1^Department of Psychiatry and Psychotherapy, Klinikum rechts der Isar der Technischen Universität München, Munich, Germany; ^2^Department of Diagnostic and Interventional Neuroradiology, Klinikum rechts der Isar der Technischen Universität München, Munich, Germany; ^3^Department of Psychiatry, University Hospital of Rion, University of Patras, Rion Patras, Greece; ^4^Department of Nuclear Medicine, Klinikum Bayreuth, Bayreuth, Germany; ^5^Department of Psychiatry and Psychotherapy, Ludwig-Maximilians-Universität München, Munich, Germany

**Keywords:** Alzheimer’s disease, mild cognitive impairment, Alzheimer’s dementia, fMRI, intrinsic functional connectivity, amygdala

## Abstract

Very early Alzheimer’s disease (AD) – i.e., AD at stages of mild cognitive impairment (MCI) and mild dementia – is characterized by progressive structural and neuropathologic changes, such as atrophy or tangle deposition in medial temporal lobes, including hippocampus and entorhinal cortex and also adjacent amygdala. While progressively disrupted intrinsic connectivity of hippocampus with other brain areas has been demonstrated by many studies, amygdala connectivity was rarely investigated in AD, notwithstanding its known relevance for emotion processing and mood disturbances, which are both important in early AD. Intrinsic functional connectivity (iFC) patterns of hippocampus and amygdala overlap in healthy persons. Thus, we hypothesized that increased alteration of iFC patterns along AD is not limited to the hippocampus but also concerns the amygdala, independent from atrophy. To address this hypothesis, we applied structural and functional resting-state MRI in healthy controls (CON, *n* = 33) and patients with AD in the stages of MCI (AD-MCI, *n* = 38) and mild dementia (AD-D, *n* = 36). Outcome measures were voxel-based morphometry (VBM) values and region-of-interest-based iFC maps of basolateral amygdala, which has extended cortical connectivity. Amygdala VBM values were progressively reduced in patients (CON > AD-MCI and AD-D). Amygdala iFC was progressively reduced along impairment severity (CON > AD-MCI > AD-D), particularly for hippocampus, temporal lobes, and fronto-parietal areas. Notably, decreased iFC was independent of amygdala atrophy. Results demonstrate progressively impaired amygdala intrinsic connectivity in temporal and fronto-parietal lobes independent from increasing amygdala atrophy in very early AD. Data suggest that early AD disrupts intrinsic connectivity of medial temporal lobe key regions, including that of amygdala.

## Introduction

Alzheimer’s disease (AD) is a neurodegenerative disease, which accounts approximately for two-thirds of dementia in old age (1). It is well established that neuropathological signs of neurodegeneration, such as neurofibrillary tangles and cell loss, are detectable in the medial temporal lobes (MTL) at early disease stages even before symptom onset, already (2, 3). Neuropathological changes in the hippocampus appear to be associated with impairment of declarative memory (4), which often represents one of the first symptoms of AD, already present at the stage of mild cognitive impairment (MCI) (5, 6). Very early stages as used throughout the manuscript entail early symptomatic stages of AD fulfilling the criteria of the National Institute on Aging – Alzheimer’s Association for probable AD (7) or MCI due to AD (8). In addition, *in vivo* imaging assessment such as resting-state functional MRI (rs-fMRI) demonstrated increasingly disrupted intrinsic functional connectivity (iFC, i.e., synchronized ongoing activity) of the hippocampus with cortical iFC networks, such as the default-mode network (9–11). Beyond the hippocampus, careful analysis of post-mortem findings demonstrates that, even in early prodromal stages of the disease, adjacent parts of the amygdala are affected as well (2, 12, 13), and imaging studies consistently found atrophy of the amygdala in MCI and AD dementia (AD-D) (14, 15). These findings suggest that amygdala connectivity might be disrupted by AD, too.

Indeed, one recent study has provided first evidence for impaired amygdala iFC in MCI and AD-D (16). The authors of this study also reported progression of amygdala iFC impairments over time in a small subsample of 13 MCI patients. However, Yao and colleagues did not address whether amygdala iFC disruption might be influenced by potential amygdala atrophy. iFC is related to the structural configuration (17), which should, therefore, be considered as a relevant factor of disrupted iFC. Thus, further evidence is required to confirm impaired amygdala iFC in different stages of AD and to determine whether alterations remain significant after controlling for structural volume decreases.

To address this question, we used structural and rs-fMRI in healthy subjects (CON), patients with MCI due to AD (AD-MCI), and AD-D. We specified our analysis to the basolateral amygdala subdivision, which is tightly coupled to hippocampus and temporal regions and characterized by extended cortical iFC pattern, including areas early affected by AD (18). Outcome measures were voxel-based morphometry (VBM) values and region-of-interest (ROI)-based functional connectivity maps of the amygdala, reflecting global brain and amygdala volumes and iFC, respectively. Statistical group contrasts of iFC maps were corrected for amygdala volume, age, total brain volume, and sex. We hypothesized progressively reduced amygdala iFC in AD-MCI and AD-D, independently from amygdala atrophy.

## Materials and Methods

### Subjects

Thirty-three healthy controls (CON), 38 patients diagnosed with AD-MCI, and 36 patients with AD-D participated in the study. A summary of subjects’ demographics and relevant clinical information is listed in Table 1. All participants were recruited from the memory clinic of the Department of Psychiatry, Klinikum rechts der Isar, Technische Universität München (TUM), Munich, Germany. Subjects provided written-informed consent before participating in the study, which was approved by the Ethics Commission at the TUM. For all participants clinical examinations included interviews, in the case of patients also with an informant as well as psychiatric, neurological, and physical examinations, structural MRI, and routine laboratory blood tests. Psychometric evaluation was based on the Neuropsychological Assessment Battery of the Consortium to Establish a Registry for Alzheimer’s Disease (CERAD-NAB) (19). Clinical diagnosis was established by consensus in a multidisciplinary team. All patients fulfilled National Institute on Aging – Alzheimer’s Association criteria for probable AD (7) or MCI due to AD with decreased Aβ42 in cerebral spinal fluid (CSF), and/or reduced glucose metabolism in the temporoparietal cortex on FDG-PET (8). Exclusion criteria were severe white matter (WM) hyperintensities, other major neurological conditions, such as other types of dementia (e.g., vascular dementia, Lewy bodies), other neurodegenerative diseases (e.g., Huntington’s disease, fronto-temporal lobe degeneration), stroke, brain tumors, and other systemic diseases that affect brain function. Furthermore, patients had to be free of current clinical symptoms of psychiatric disorders as assessed by a psychiatrist (anxiety, major depression). Patients with previous depressive symptoms who did not meet criteria for a major depressive episode under medication were allowed in the study. In the respective groups (AD-D/AD-MCI/CON), 16/16/8 participants were treated for hypertension (beta-blockers, ACE-inhibitors, calcium channel blockers), 14/10/11 for hypercholesterolemia (statins), 3/1/1 individuals were diagnosed with diabetes mellitus, and 6/5/0 received antidepressant medication (mirtazapine, escitalopram, fluoxetine). Four patients diagnosed with AD-MCI and all patients with AD-D received cholinesterase inhibitors; except for two that were treated with the glutamatergic *N*-Methyl-d-aspartate receptor (NMDAR) antagonist memantine. Controls were free of any psychotropic medication, did not show psychometric (CERAD-NAB) deficits, and CDR score was 0.

**Table 1:** **Demographic and psychometric details from CERAD-NAB of study groups**.

Characteristic	CON	AD-MCI	AD-D	*F*	Sig.
			
	M (SD)	M (SD)	M (SD)		
Age	56.15 (9.31)	59.61 (9.34)	62.86 (8.90)*	4.60	0.012
Education	10.67 (1.71)	10.52 (1.94)	9.48 (2.82)	2.21	0.117
% male	36.40	55.30	50.00	2.62^a^	0.207
CERAD total score	89.88 (7.84)	67.50 (13.79)*	49.49 (17.49)*^§^	74.21	0.000
MMSE	29.44 (0.78)	27.00 (1.93)*	21.91 (5.57)*^§^	43.10	0.000
Verbal fluency	23.00 (1.95)	15.53 (6.47)*	11.43 (6.98)*^§^	36.27	0.000
Modified BNT	14.72 (0.32)	13.81 (1.56)	11.66 (4.27)*^§^	12.28	0.000
Word list learning	23.28 (3.12)	15.81 (4.52)*	10.00 (6.28)*^§^	64.68	0.000
Constructional praxis	10.56 (0.87)	10.11 (1.54)	8.26 (2.67)*	15.08	0.000
Word list recall	8.78 (1.32)	4.31 (3.53)*	1.94 (2.21)*^§^	62.40	0.000
Word list recognition	9.94 (0.36)	8.17 (2.16)*	7.85 (2.78)*	10.05	0.000

*Score differs significantly from CON group (*p* < 0.05).

*^§^Score differs significantly from MCI group (*p* < 0.05)*.

*^a^χ^2^ value calculated with Kruskal–Wallis test*.

*CON, healthy controls; AD-MCI, mild cognitive impairment due to Alzheimer’s disease; AD-D, dementia due to Alzheimer’s disease; CERAD, Consortium to Establish a Registry for Alzheimer’s disease; MMSE, Mini–Mental Status Exam; Modified BNT, Modified Boston Naming Test*.

All participants underwent 10 min of resting-state fMRI and about 5 min of structural MRI. For fMRI, each participant was instructed simply to keep her/his eyes closed, not to think of anything particular and not to fall asleep. We verified that subjects stayed awake by communicating with them *via* intercom immediately after the fMRI scan.

### MRI Data Acquisition

Structural and functional MRI of the brain was obtained using standard procedures as described previously (20). Imaging was performed on a 3-T whole body MR scanner (Achieva, Philips, Netherlands) using an 8-channel phase-array head coil. T1-weighted structural data were obtained using a magnetization-prepared rapid acquisition gradient echo sequence (TE = 4 ms, TR = 9 ms, TI = 100 ms, flip angle = 5°, FoV = 240 mm × 240 mm, matrix = 240 × 240, 170 slices, voxel size = 1 mm × 1 mm × 1 mm). Whole-brain fMRI data were collected by using a gradient EPI sequence (TE = 35 ms, TR = 2000 ms, flip angle = 82°, FoV = 220 mm × 220 mm, matrix = 80 × 80, 32 slices, slice thickness = 4 mm, 0 mm interslice gap; 10 min scan duration resulting in 300 volumes).

### Data Analysis

#### Voxel-Based Morphometry

In order to determine structural changes associated with MCI and AD-D, we performed VBM following a previously described protocol (21). The VBM12 toolbox for SPM^1^ was used for data preprocessing and analysis. Structural images were corrected for bias-field inhomogeneity and registered using linear (12-parameter affine) and non-linear transformations. Tissue classification into gray matter (GM), WM, and CSF was performed within the same generative model (22). GM images were modulated to account for volume changes based on the normalization process. We only considered non-linear volume changes so that subsequent analyses were not impacted by differences in head size. Images were spatially normalized into the stereotactic space of the Montreal Neurological Institute (MNI) and smoothed with an 8 mm (FWHM) Gaussian kernel (final voxel size: 1 mm × 1 mm × 1 mm). Further analyses were restricted to voxels with an *a priori* GM probability of >0.1 to avoid borderline effects between GM and WM. In order to assess whole-brain volumetric GM group differences, we applied a voxel-wise SPM8 ANOVA with maps of segmented GM images derived from a VBM analysis. Group differences were assessed *via* two-sample *t*-tests (*p* < 0.05 family-wise error (FWE) corrected, extent threshold: 20 voxels). Furthermore, focused VBM analysis of amygdala was restricted to ROI templates based on probabilistic cytoarchitectonic maps of basolateral amygdala as implemented in the Anatomy Toolbox for SPM (302 and 324 voxels for left and right amygdala, respectively). Subsequent two-sample *t*-tests were Bonferroni-corrected.

#### Seed-Based iFC Analysis

For each subject, the first three functional scans were discarded due to magnetization effects. SPM8 (Wellcome Department of Cognitive Neurology, London) was used for motion correction, spatial normalization into the stereotactic space of the Montreal Neurological Institute (MNI), and spatial smoothing (8 mm × 8 mm × 8 mm Gaussian kernel, final voxel size: 3 mm × 3 mm × 3 mm). To account for potential motion-induced artifacts, temporal signal-to-noise ratio (tSNR) and point-to-point head motion were estimated for each subject (23). Excessive head motion (cumulative motion translation or rotation >3 mm or 3° and mean point-to-point translation or rotation >0.15 mm or 0.1°) was applied as exclusion criterion. Point-to-point motion was defined as the absolute displacement of each brain volume compared to its previous volume. None of the participants had to be excluded. ANOVA yielded no significant differences between groups in mean point-to-point translation or rotation in any direction (*p* > 0.30) or in tSNR (*p* > 0.30). Furthermore, we assessed framewise displacement to characterize head movement in our subjects (24). No significant group differences were detected in mean framewise displacement. For seed-based iFC analysis, regions-of-interest (ROIs) were chosen corresponding to bilateral basolateral amygdala as implemented in the Anatomy Toolbox for SPM, resulting in ROIs of 88 and 103 voxels for left and right amygdala, respectively (25). ROI were transformed for analysis by use of MarsBaR (Release 0.42,^2^). After Butterworth bandpass-filtering of all voxel time courses for the frequency range from 0.009 to 0.08 Hz, we extracted voxel time courses of seed ROIs and reduced them to ROI-representative time courses by singular value decomposition, respectively. Each time course was entered into a first-level fixed-effects general linear model in SPM8, and two separate iFC analyses (i.e., left amygdala, right amygdala) were performed for each subject. For each model, additional regressors for global GM, WM, CSF BOLD-signal, and six movement parameters (three translational and three rotational directions) were included as covariates-of-no-interest. Thus, individual iFC maps were obtained for left and right amygdala separately.

Group level analyses were performed using a flexible factorial model of analysis of variance (ANOVA) within SPM8 that included covariates-of-no-interest: sex, age, and total brain matter volume. To account for amygdala volume and atrophy, individual amygdala VBM values (separately for left and right amygdala, respectively) were calculated, and – in a second model – included as covariates-of-no-interest. Factors were group (with levels CON, AD-MCI, and AD-D) and ROI (with levels left and right amygdala). To increase sensitivity, both ANOVA models were restricted to explicit masks at a lenient statistical threshold based on the conjunction of positive and negative group iFC maps (one-sample *t*-tests *p* < 0.05, uncorrected). The parameter of interest for both ANOVA models was the main effect of group, and appropriate *post hoc t*-tests were applied in order to reveal the direction of change. Due to clarity of presentation, we only display results regardless of hemispheres. Statistical thresholds were set to *p* < 0.05, FWE corrected, height threshold *p* < 0.005. Reported voxel coordinates correspond to standardized MNI space.

## Results

### Atrophy in Basolateral Amygdala in Very Early AD

**Figure 1:**
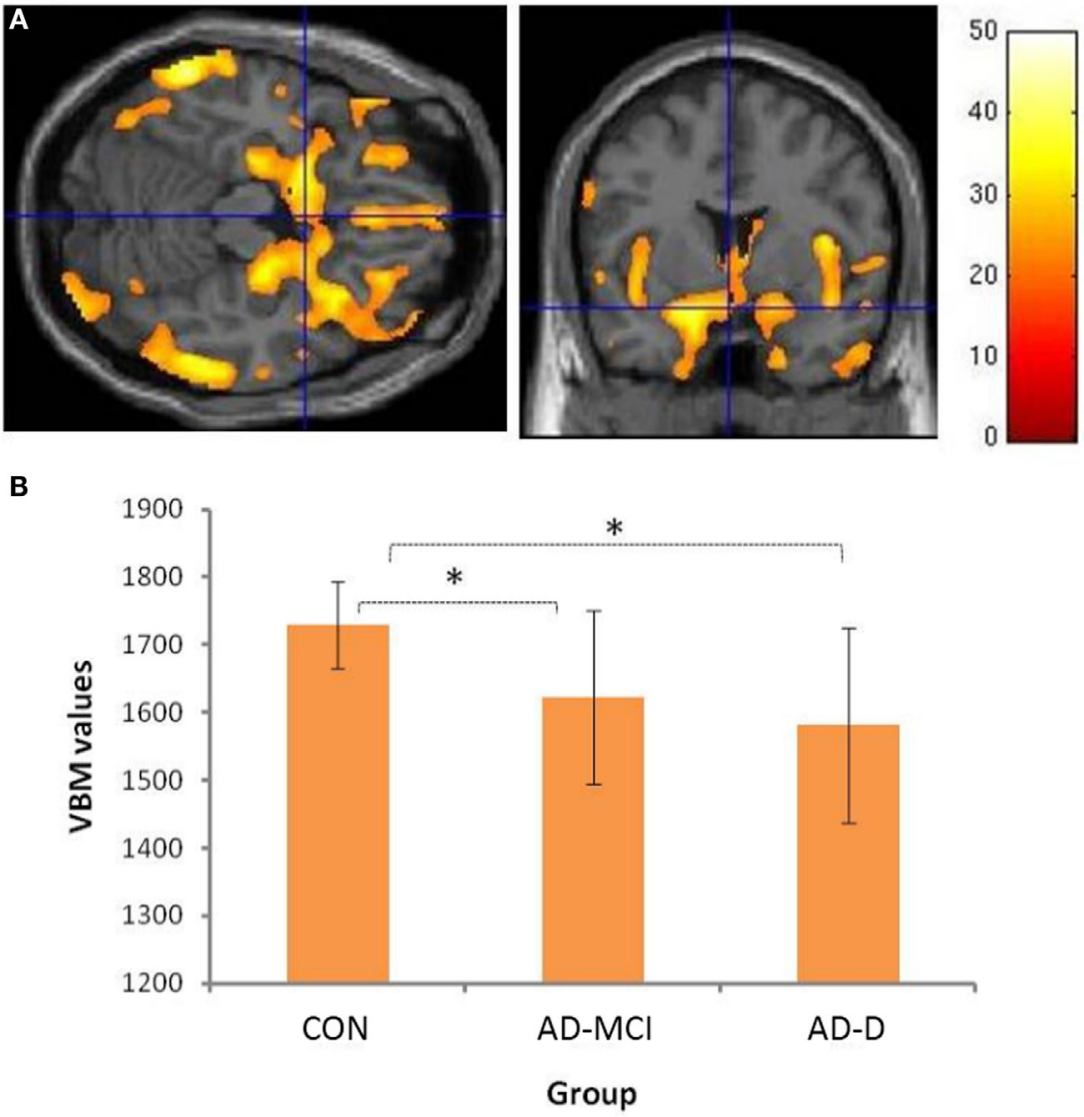
**Volumetric group differences across healthy controls (CON), patients with mild cognitive impairment due to Alzheimer’s disease (AD-MCI), and patients with dementia due to Alzheimer’s disease (AD-D)**. **(A)** shows the main effect of groups with respect to atrophy in gray matter regions; Bar represents *T*-values; *p* < 0.05, FWE corrected. Please cf. to Table S1 in Supplementary Material for details of involved clusters. **(B)** The *y*-axis denotes VBM values of amygdala (mean of bilateral ROI voxels) for groups depicted on *x*-axis. Asterisks indicate significant differences, Bonferroni-corrected *p* values: CON–AD-MCI: *p* = 0.001; CON–AD-D: *p* < 0.001; AD-MCI–AD-D: *p* = 0.422. Abbreviations: CON, healthy controls; AD-MCI, patients with mild cognitive impairment due to Alzheimer’s disease; AD-D, patients with dementia due to Alzheimer’s disease.

Group comparisons revealed decreased amygdala gray matter volumes in AD-MCI and AD-D relative to CON (Figure 1B). Slightly lower amygdala volumes in AD-D than in AD-MCI were not significant in the direct contrast. Beyond amygdala, voxel-wise ANOVA revealed a typical pattern of atrophy in AD, including medial and lateral temporal lobes, parieto-occipital regions, insula and cerebellum (Figure 1A and Table S1 in Supplementary Material).

### Aberrant Amygdala iFC in Very Early AD

**Figure 2:**
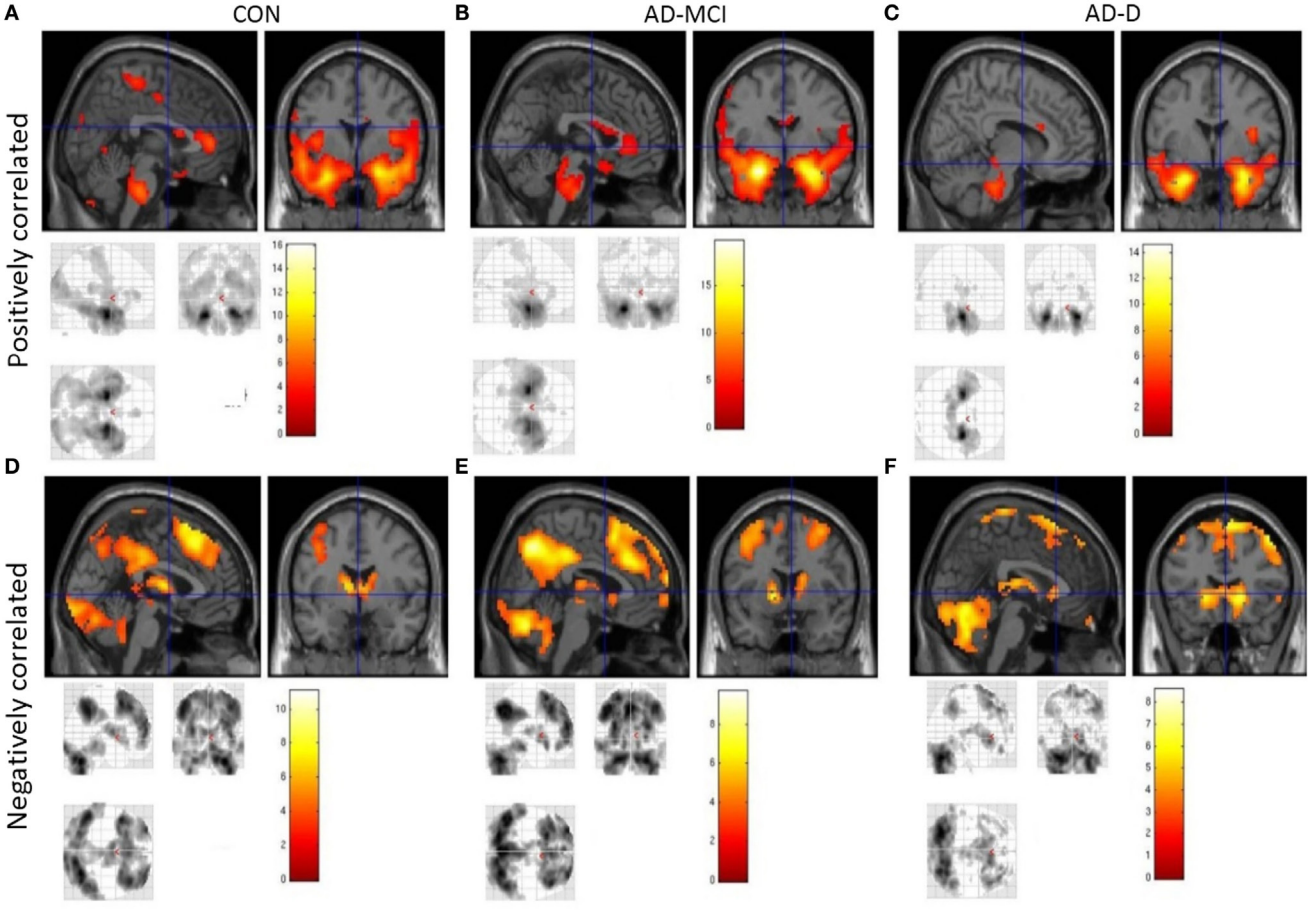
**Intrinsic whole-brain functional connectivity patterns of amygdala across healthy controls (CON), patients with mild cognitive impairment due to Alzheimer’s disease (AD-MCI) and patients with dementia due to Alzheimer’s disease (AD-D)**. Images **(A–C)** show positively correlated activity across groups; images **(D–F)** show negatively correlated activity across groups as labeled. Displayed are clusters larger than 20 voxels, bars represent *T*-values, *p* < 0.05, FWE corrected. Abbreviations: CON, healthy controls; AD-MCI, patients with mild cognitive impairment due to Alzheimer’s disease; AD-D, patients with dementia due to Alzheimer’s disease.

In healthy controls, positive amygdala iFC covered frontal cortical regions, including the anterior cingulate cortex (ACC) and insular cortex, as well as medial and lateral temporal regions, somatosensory cortex, small regions of parietal, and occipital cortex. Negative amygdala iFC covered dorsomedial and lateral frontal regions, medial parietal and lateral occipital regions, as well as caudate, thalamus, and cerebellum. In patients with AD-MCI, the iFC pattern was similar to those of healthy controls, despite few regions dropping below significance after correction for multiple testing (e.g., posterior cingulate cortex). In AD-D, additional regions did not emerge as significant (e.g., ACC). However, overall, our results indicate relatively preserved amygdala connectivity in patients (Figure 2, Tables S2–S7 in Supplementary Material).

**Figure 3:**
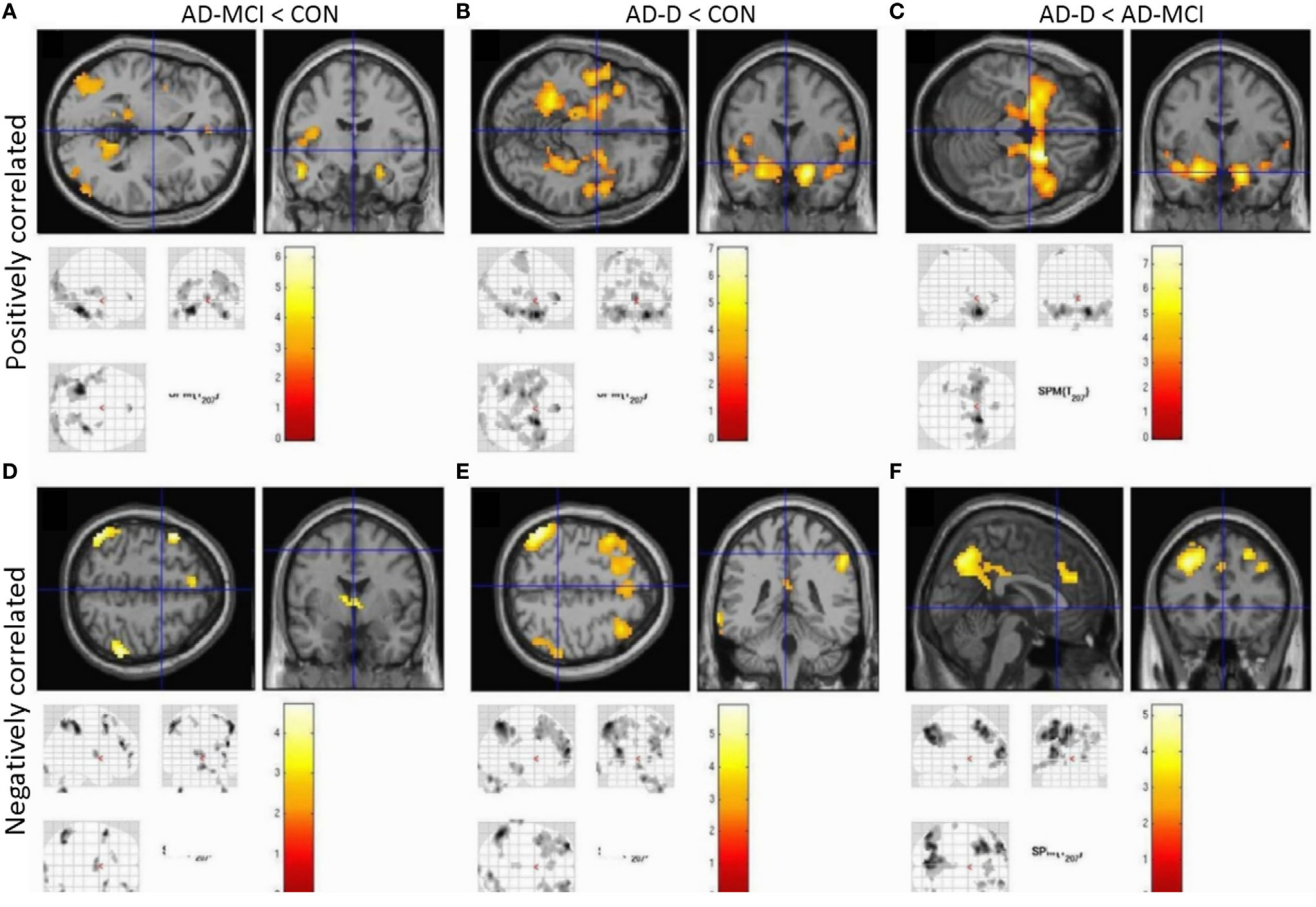
**Group contrasts of amygdala whole-brain functional connectivity, taking volumetric amygdala differences into account by including individual VBM scores as covariates into ANOVA models**. Upper row **(A–C)** shows positively correlated activity, lower row **(D–F)** anticorrelated activity. Columns code different group contrasts: **(A,D)** CON over AD-MCI; **(B,E)** CON over AD; **(C,F)** AD-MCI over AD-D. Bars represent *T*-values, *p* = 0.05, FWE corrected, cluster size ≥ 20 voxels. Abbreviations: CON, healthy controls; AD-MCI, patients with mild cognitive impairment due to Alzheimer’s disease; AD-D, patients with dementia due to Alzheimer’s disease.

**Figure 4:**
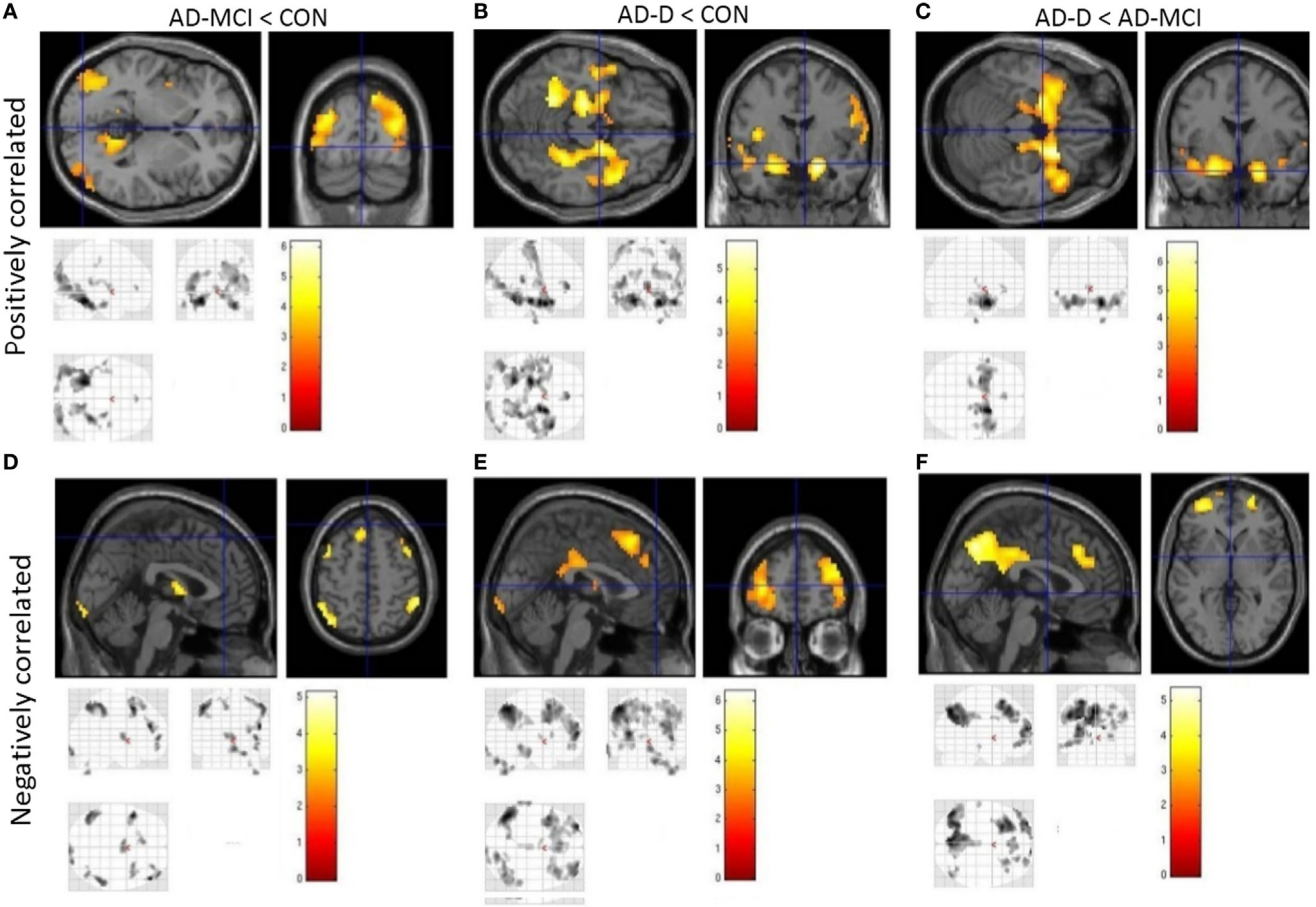
**Group contrasts of amygdala whole-brain functional connectivity, without taking volumetric amygdala differences into account**. Upper row **(A–C)** shows positively correlated activity, lower row **(D–F)** anticorrelated activity. Columns code different group contrasts: Figures 3A,D CON over AD-MCI; Figures 3B,E CON over AD-D; Figures 3C,F AD-MCI over AD-D. Bars represent *T*-values, *p* = 0.05, FWE corrected, cluster size ≥ 20 voxels. Abbreviations: CON, healthy controls; AD-MCI, patients with mild cognitive impairment due to Alzheimer’s disease; AD-D, patients with dementia due to Alzheimer’s disease.

Concerning group comparisons, in the following, we report detailed results of the ANOVA model including amygdala VBM values as covariate-of-no-interest (Figure 3). In Figure 4, results of the model without amygdala volume values are depicted. Results of both models are largely similar. In general, positive and negative amygdala iFC is progressively disrupted in patients i.e., we observe the pattern CON > AD-MCI, CON > AD-D, and AD-MCI > AD-D (Figure 3, Tables S8–S13 in Supplementary Material). Reduced positive amygdala iFC in AD-MCI in comparison to CON was found in parieto-occipital regions, insula, and hippocampus (Figure 3A). In AD-D, in comparison to CON, regions in anterior insula, parahippocampal gyrus, hippocampus, temporal pole, and medial prefrontal cortex were additionally decoupled in patients (Figure 3B). In line with this finding, AD-D patients had reduced amygdala iFC mainly in medial temporal and insular regions in comparison to AD-MCI patients (Figure 3C). Reduced negative amygdala iFC was detected in AD-MCI patients relative to CON in lateral parietal cortices, dorsolateral and medial prefrontal cortices, as well as thalamus (Figure 3D). In AD-D vs. CON, similar clusters were found but with larger extent (Figure 3E). Direct comparison between AD-D and AD-MCI confirms this finding by revealing reduced negative amygdala iFC in medial and lateral parietal and prefrontal cortices (Figure 3F). Increased iFC in patients did not remain significant after appropriate statistical correction for multiple testing.

## Discussion

The current study investigated basolateral amygdala iFC in very early AD at the stage of MCI. With increased clinical severity of the disease, i.e., AD-MCI and AD-D, progressive loss of amygdala iFC was observed, independently from amygdala volume loss. Together with known hippocampus iFC loss, results indicate that early AD disrupts intrinsic connectivity of medial temporal lobe key regions.

### Bilateral Amygdala Atrophy in Very Early AD

We found bilateral amygdala atrophy in patients with AD-MCI and AD-D, respectively (Figure 1). This finding replicates previous imaging results (14, 15, 26, 27). Furthermore, our data are consistent with findings by Markesbery and colleagues comprising longitudinal clinicopathological cohort studies (12, 13) and indicating neurofibrillary pathology and neuritic plaques in amygdala of patients. Moreover, lower beta-amyloid (Aβ-42) levels in CSF were associated with amygdala atrophy rates, even in cognitively normal subjects (28). Taken together, our structural finding of reduced amygdala volumes adds to previous literature, indicating early neurodegeneration in very early AD.

### Progressively Disrupted Amygdala iFC Independent from Amygdala Atrophy

In healthy controls, observed positive and negative iFC of amygdala with a number of regions (Figure 2), including the anterior cingulate cortex (ACC), insula, and temporal and parietal regions for positive iFC and medial and lateral parietal and prefrontal cortices for negative iFC, matches previously reported amygdala connectivity patterns, indicating the reliability of our approach (18, 20, 29). Progressively reduced iFC in patient groups, mainly in occipital, insular, and medial temporal regions for positive iFC (Figure 4), is in line with previous findings (30). Beyond prior studies, we demonstrate that also amygdala negative iFC in medial and lateral parietal and prefrontal cortices is progressively reduced in patients (Figure 4). Furthermore, we suggest that disrupted amygdala iFC is independent from amygdala atrophy (Figures 3 and 4). However, it needs to be taken into account that further studies are required to examine structural integrity of amygdala-cortical tracts and clarify the distinct relation between functional connectivity, localized atrophy, and atrophic tracts of the amygdala. Moreover, the detailed interaction between regions across different groups including switches between positive and negative interregional connectivity may further increase our understanding of the dynamic interplay, which may be assessed by future studies. Taken together, our results demonstrate progressive and extended amygdala iFC disruption in AD even at the stage of MCI, which affects large parts of the cortex and which is of functional nature and is not explained sufficiently by amygdala atrophy.

### Methodological Issues, Clinical, and Neuropsychological Implications

Our finding of disrupted amygdala iFC includes negative iFC based on standard seed-based iFC analysis. In particular, our analysis procedure included global signal regression to get rid of physiological noise (e.g., respiratory- and cardiac-based signals) from ongoing fMRI signal at rest. However, several previous studies demonstrated that global signal regression induces – under several conditions – artificial BOLD correlations, particularly negative correlations i.e., negative iFC, suggesting careful use of global signal regression and negative iFC (31–34). We decided to use global signal regression for the following reasons: (i) recent studies provided empirical evidence for the biological origin of negative BOLD iFC independent from global signal regression (31, 35). (ii) Particularly for the AY, negative iFC with frontolimbic areas was reported for awake rats, again independent of global signal regression (36). This finding indicates robustness of amygdala negative iFC across species, as well as its independence from global signal regression. (iii) Previous studies in humans used global signal regression when investigating amygdala iFC with results largely comparable to those found in animals (29). (iv) Recent models of negative iFC suggest that anticorrelations emerge as a functional consequence of multiple indirect anatomical connections and temporal delays (37). As a limitation, we did not control for the effect that cardiovascular and antidepressant medication might have on BOLD signal and iFC of the amygdale (38–40). While current depressive symptoms were an exclusion criterion, six patients of the AD-D group and five patients of the MCI-AD group were treated with antidepressant drugs. We cannot finally rule out, that the observed effects are impacted by depressive disorder, even if currently asymptomatic. Furthermore, despite taking local amygdala atrophy into account, we cannot determine the relation of iFC changes with atrophy of structural amygdalo-cortical tracts by the applied measures. Future studies e.g., employing diffusion tensor imaging will help to clarify this.

Amygdala and its intrinsic connectivity are involved in emotion and mood processing. Impaired emotion and mood processing and depressive symptoms are frequently observed in early AD (41, 42) and often precede the onset of dementia (43). Previous studies suggested that dysfunction of neuronal networks which include both the hippocampus and the amygdala may play a major role in the pathogenesis of depressive symptoms (20, 44). On the other hand, also cognitive deficits in late-onset depression were shown to be related to aberrant amygdala connectivity (45). Moreover, Poulin et al. found a relation between amygdala atrophy and anxiety, which is another common behavioral symptom in AD (41, 46). Together, these data suggest that impaired amygdala iFC might be involved in the risk for depressive symptoms in early AD. Unfortunately clinical-neuropsychological data from our patients about their state of depressive symptoms were unsuitable to test this idea in detail. Nevertheless, we emphasize the importance of screening for depressive symptoms in patients with cognitive impairment and for considering neurodegeneration when depressive patients do not respond to medication as expected or when they have an unusual profile in neuropsychological assessments. Future studies are necessary to study distinct key regions of MTL including amygdala for their relevance for depressive symptoms in early AD.

## Conclusion

To our knowledge, our data provides first evidence for a progressively disrupted iFC of the basolateral amygdala in very early AD, which is independent of co-occurring amygdala atrophy. Data underline that broad amygdala alterations are an early sign of AD.

## Author Contributions

All others approved the final version and agree to be accountable for all aspects of the work. In addition the individual authors contributed as follows: MO: conception of the work, analysis and interpretation of data, and drafting the work; LP: acquisition of data and revising work for intellectual content; PA, SF: acquisition and analysis of data and revising work for intellectual content; TG, JD-S, AK, HF, and CZ: interpretation of data and revising it critically for intellectual content; AW, CS: conception and design of experiments, interpretation of data, revising work critically for important intellectual content; HP: conception of work, acquisition, analysis and interpretation of data, drafting the work. MB: Acquisition and analysis of data and drafting the work.

## Conflict of Interest Statement

All authors, i.e., Dr. MO, LP, MB, Dr. PA, Dr. TG, Dr. SF, Prof. JD-S, Prof. Dr. AK, Prof. Dr. HF, Prof. Dr. CZ, Dr. AW, Dr. CS, and Dr. HP reported no biomedical financial interests or potential conflicts of interest relevant to the subject matter of the manuscript.

## Footnotes

^1^http://dbm.neuro.uni-jena.de/vbm.html

^2^http://marsbar.sourceforge.net/
