# Supplementary material for: Progressively Disrupted Intrinsic Functional Connectivity of Basolateral Amygdala in Very Early Alzheimer’s Disease
Source: Front Neurol. 2016 Sep 19;7:132. doi: 10.3389/fneur.2016.00132 (PMC5027206; doi:10.3389/fneur.2016.00132)
Supplement: Supplementary file 1 [file data_sheet_1.docx]

# Supplementary material

## Supplementary Tables S1-13

| Table S1. Brain regions showing significant reduction of gray matter mass based on VBM (ANOVA, main effect of group). | | | | | | | | |
| --- | --- | --- | --- | --- | --- | --- | --- | --- |
| **Lobe** | **Anatomy** | **Laterality** | **Cluster size** | **x** | **y** | **z** | **(z)** | **p(FWE)** |
| Frontal | Anterior PFC | L | 1312 | -22 | 65 | 1 | 6,35 | <,001 |
|  | Medial orbital gyrus |  | 14967 | 0 | 61 | -15 | 6,83 | <,001 |
|  | Medial frontal gyrus | R |  | 2 | 36 | -12 | 6,94 | <,001 |
|  | ACC | L |  | -2 | 27 | 27 | 6,72 | <,001 |
|  | Inferior frontal gyrus | L | 2699 | -50 | 31 | -15 | 6,9 | <,001 |
|  | DLPFC | R | 372 | 55 | 29 | 23 | 6,43 | <,001 |
|  | Orbitofrontal | R | 1282 | -26 | 42 | -18 | 6,36 | <,001 |
| Temporal | Superior temporal gyrus | L | 143 | -56 | 5 | -6 | 6,18 | <,001 |
|  | Middle temporal gyrus | R | 158033 | 53 | -23 | -5 | 7,56 | <,001 |
|  | Parahippocampal gyrus | R |  | 24 | -15 | -15 | 7,05 | <,001 |
|  |  | L | 123 | -25 | -14 | -15 | 6,76 | <,001 |
|  | HP | L | 33 | -25 | -16 | -17 | 6,64 | <,001 |
|  |  | R | 33 | 27 | -16 | -17 | 6,73 | <,001 |
|  | Amygdala | L | 33 | -13 | -1 | -15 | 6,95 | <,001 |
|  |  | R | 33 | 19 | 0 | -16 | 6,45 | <,001 |
| Insula | Insular cortex | L | 123 | 46 | -12 | 5 | 7,33 | <,001 |
|  |  | R | 123 | -44 | -14 | 6 | 6,44 | <,001 |
| Parietal | PCC | R | 118 | 1 | -34 | 42 | 6,2 | <,001 |
|  | Precuneus | R |  | 1 | -62 | 39 | 6,48 | <,001 |
|  | Supramarginal gyrus | L | 63 | -66 | -47 | 31 | 6,39 | <,001 |
|  |  | R | 123 | 65 | -45 | 23 | 6,75 | <,001 |
| Occipital | Cuneus | R | 123 | 1 | -89 | 25 | 6,16 | <,001 |
|  | Middle occipital gyrus | L | 158033 | -52 | -76 | -8 | <8 | <,001 |
|  |  | R |  | 53 | -80 | 5 | 6,61 | <,001 |
|  | Associative visual c. | R |  | 45 | -79 | 34 | 7,63 | <,001 |
|  |  | L |  | -50 | -77 | -9 | 7,82 | <,001 |
| Cerebellum | Cerebellar tonsil | R | 33 | 38 | -49 | -55 | 5,79 | <,001 |
| PFC = prefrontal cortex; ACC = anterior cingulated cortex; DLPFC = dorsolateral prefrontal cortex; PCC = posterior cingulated cortex; c. = cortex; x, y, z = coordinates of primary peak locations in standard MNI space. For clusters with more than one peak, local maxima are listed. Cluster size in voxels. | | | | | | | | |

| Table S2. List of brain regions showing positively correlated activity with amygdala in healthy controls. | | | | | | | | |
| --- | --- | --- | --- | --- | --- | --- | --- | --- |
| **Lobe** | **Anatomy** | **Laterality** | **Cluster size** | **x** | **y** | **z** | **(z)** | **p(FWE)** |
| Frontal | ACC |  | 114 | 0 | 44 | 7 | 5,84 | <0,001 |
|  | Precentral g., motor c. | R | 38 | 30 | -19 | 76 | 4,27 | <0,001 |
| Temporal | Amygdala | L | 5736 | -21 | -7 | -20 | <8 | <0,001 |
|  | HP, Parahippoc. | L |  | -25 | -8 | -32 | <8 | <0,001 |
|  | Middle temporal gyrus | L |  | -51 | 8 | -17 | <8 | <0,001 |
|  |  | R | 5025 | 54 | 8 | -17 | <8 | <0,001 |
|  | Amygdala | R |  | 21 | -7 | -17 | <8 | <0,001 |
|  | HP, Parahippoc. | R |  | 27 | -7 | -32 | <8 | <0,001 |
| Insula | Insula | R | 37 | 45 | -28 | 16 | 3,96 | <0,001 |
|  |  | L | 37 | -39 | -34 | 16 | 6,26 | <0,001 |
| Parietal | PCCdorsal | L | 283 | -6 | -13 | 49 | 4,12 | <0,001 |
|  | Suplementary motor a. | L |  | -3 | -28 | 61 | 4,76 | <0,001 |
|  |  | R |  | 6 | -31 | 61 | 4,59 | <0,001 |
|  | Prima ry somatosensory c. | L |  | -45 | -22 | 58 | 4,3 | <0,001 |
|  |  | R |  | 45 | -22 | 58 | 4,77 | <0,001 |
|  | Postcentralgyrus | R | 31 | 21 | -40 | 79 | 3,87 | <0,001 |
| Occipital | inferior occipital | L | 5736 | -12 | -100 | 10 | 3,11 | 0,001 |
|  | Middle occipital | R | 5025 | 33 | -97 | 13 | 4,62 | <0,001 |
|  |  | L |  | -15 | -94 | 31 | 5,62 | <0,001 |
| Brainstem | Pons |  | 114 | 0 | -28 | -38 | 6,34 | <0,001 |
| ACC = anterior cingulated cortex; PCC = posterior cingulated cortex; c.= cortex; a. = area; x, y, z = coordinates of primary peak locations in standard MNI space. For clusters with more than one peak, local maxima are listed. Cluster size in voxels. | | | | | | | | |

| Table S3. List of brain regions showing positively correlated activity with amygdala in AD-MCI. | | | | | | | | |
| --- | --- | --- | --- | --- | --- | --- | --- | --- |
| **Lobe** | **Anatomy** | **Laterality** | **Cluster size** | **x** | **y** | **z** | **(z)** | **p(FWE)** |
| Frontal | ACC |  | 196 | 0 | 32 | -2 | 4,95 | <0,001 |
| Temporal | Amygdala | L | 6394 | -24 | -4 | -23 | <8 | <0,001 |
|  |  | R |  | 24 | -4 | -23 | <8 | <0,001 |
|  | HP, pHP | R |  | 30 | -4 | -23 | <8 | <0,001 |
|  |  | L |  | -27 | -4 | -26 | <8 | <0,001 |
|  | Temporopolar area | R |  | 48 | 17 | -20 | <8 | <0,001 |
|  |  | L |  | -30 | 14 | -35 | 7,11 | <0,001 |
|  | Superior temporal | R |  | 65 | -7 | 7 | 4,53 | <0,001 |
|  |  | L |  | -59 | -7 | 4 | 4,32 | <0,001 |
| Parietal | Motorcortex | L | 350 | -24 | -52 | 73 | 5,41 | <0,001 |
|  | Primary motor | L |  | -24 | -28 | 61 | 4,11 | <0,001 |
|  |  | R |  | 24 | -31 | 61 | 4,07 | <0,001 |
|  | Primarysomatosensory | L |  | -42 | -28 | 70 | 4,37 | <0,001 |
| Occipital | Inferior occipital | L | 89 | -9 | -103 | 13 | 5,54 | <0,001 |
|  |  | R |  | 18 | -103 | 16 | 4,33 | <0,001 |
| Brainstem | Pons |  | 6394 | 0 | -28 | -41 | 5,71 | <0,001 |
| AD-MCI = Mild cognitive impairment due to Alzheimer’s disease; ACC = anterior cingulated cortex; HP = hippocampus; pHP = paraHP; x, y, z = coordinates of primary peak locations in standard MNI space. For clusters with more than one peak, local maxima are listed. Cluster size in voxels. | | | | | | | | |

| Table S4. List of brain regions showing positively correlated activity with amygdala in AD-D. | | | | | | | | |
| --- | --- | --- | --- | --- | --- | --- | --- | --- |
| **Lobe** | **Anatomy** | **Laterality** | **Cluster size** | **x** | **y** | **z** | **(z)** | **p(FWE)** |
| Temporal | Medial temporal | R | 3687 | 27 | -7 | -20 | <8 | <0,001 |
|  |  | L |  | -21 | -7 | -20 | <8 | <0,001 |
|  | Frontopolar area | L |  | -39 | 14 | -35 | 5,15 | <0,001 |
|  |  | R |  | 39 | 14 | -35 | 5,57 | <0,001 |
| Insula | Insula | L |  | -45 | -10 | 19 | 4,07 | <0,001 |
|  |  | R | 24 | 36 | -19 | 19 | 4,41 | <0,001 |
| Occipital | Inferior occipital | R | 45 | 39 | -94 | 4 | 4,22 | <0,001 |
|  | Superior occipital | R |  | 36 | -91 | 19 | 3,82 | <0,001 |
|  |  | L | 33 | -39 | -88 | 19 | 4,48 | <0,001 |
| Brainstem | Pons |  | 3638 | -3 | -31 | -35 | 6,26 | <0,001 |
| AD-D = Alzheimer’s disease dementia; x, y, z = coordinates of primary peak locations in standard MNI space. For clusters with more than one peak, local maxima are listed. Cluster size in voxels. | | | | | | | | |

| Table S5. List of brain regions showing negatively correlated activity with amygdala in healthy controls. | | | | | | | | |
| --- | --- | --- | --- | --- | --- | --- | --- | --- |
| **Lobe** | **Anatomy** | **Laterality** | **Cluster size** | **x** | **y** | **z** | **(z)** | **p(FWE)** |
| Frontal | Middle frontal gyrus | L | 5259 | -30 | 59 | 13 | <8 | <,001 |
|  |  | R |  | 39 | 20 | 52 | 7,34 | <,001 |
|  | Superior frontal (FEE) | R |  | 42 | 29 | 46 | <8 | <,001 |
|  |  | L |  | -15 | 20 | 61 | 6,75 | <,001 |
|  | Premotorcortex | L |  | -3 | 21 | 63 | 6,93 | <,001 |
|  |  | R |  | 36 | 11 | 61 | 7,45 | <,001 |
| Parietal | Mid cingulate gyrus |  | 1848 | 0 | -28 | 37 | 5,96 | <,001 |
|  | PCC |  |  | 0 | -37 | 31 | 4,64 | <,001 |
|  | Angular gyrus | L |  | -51 | -55 | 43 | <8 | <,001 |
|  |  | R |  | 44 | -55 | 45 | <8 | <,001 |
|  | Supramarginal gyrus | L |  | 51 | -55 | 37 | 7,39 | <,001 |
|  |  | R |  | -51 | -55 | 34 | <8 | <,001 |
|  | Inferior parietal g. | L |  | -45 | -58 | 49 | <8 | <,001 |
|  |  | R |  | 48 | -55 | 46 | <8 | <,001 |
|  | Somatosensoryassociat. | L |  | -3 | -52 | 43 | 3,9 | <,001 |
|  | Caudate head | R | 774 | 12 | 20 | -5 | 5,3 | <,001 |
|  |  | L |  | -12 | 17 | -2 | 5,36 | <,001 |
| Temporal | Middle temporal gyrus | L | 422 | -66 | -37 | -11 | 5,59 | <,001 |
|  |  | R |  | 63 | -37 | -8 | 6,34 | <,001 |
| Occipital | Inferior occipital gyrus | R | 3947 | 27 | -91 | -17 | 7,39 | <,001 |
|  |  | L |  | -29 | -90 | -18 | 5,92 | <,001 |
| Cerebellum | Inf. semi-lunar declive | L |  | -29 | -79 | -47 | 5,17 | <,001 |
|  |  | R |  | 6 | -58 | -50 | 3,4 | <,001 |
|  | Decilve | R |  | 15 | -76 | -29 | <8 | <,001 |
|  |  | L |  | -24 | -76 | -26 | 5,77 | <,001 |
|  | Uvula | R |  | 30 | -67 | -32 | <8 | <,001 |
|  |  | L |  | -18 | -76 | -32 | 5,93 | <,001 |
| FEF = frontal eye fields; PCC = posterior cingulated cortex; g = gyrus; x, y, z = coordinates of primary peak locations in standard MNI space. For clusters with more than one peak, local maxima are listed. Cluster size in voxels. | | | | | | | | |

| Table S6. List of brain regions showing negatively correlated activity with amygdala in AD-MCI. | | | | | | | | |
| --- | --- | --- | --- | --- | --- | --- | --- | --- |
| **Lobe** | **Anatomy** | **Laterality** | **Cluster size** | **x** | **y** | **z** | **(z)** | **p(FWE)** |
| Frontal | Superior frontal gyrus | L | 5650 | -24 | 20 | 58 | <8 | <,001 |
|  |  | R |  | 27 | 23 | 58 | 5,95 | <,001 |
|  | Middle frontal gyrus | L |  | -30 | 59 | 16 | <8 | <,001 |
|  | Middle frontal gyrus (FEF) | R |  | 42 | 29 | 43 | <8 | <,001 |
|  | ACC | R | 640 | 12 | 20 | -8 | 6,08 | <,001 |
|  |  | L |  | -12 | 17 | -8 | 6,47 | <,001 |
| Parietal | Precuneus | L | 4335 | -3 | -70 | 43 | <8 | <,001 |
|  |  | R |  | 6 | -64 | 34 | 5,31 | <,001 |
|  | Somatosensory assoc. area | L |  | -3 | -58 | 46 | <8 | <,001 |
|  | PCC | L |  | -6 | -40 | 34 | 6,65 | <,001 |
|  |  | R |  | 3 | -40 | 34 | 6,49 | <,001 |
|  | Angulargyrus | L |  | -48 | -61 | 34 | <8 | <,001 |
|  |  | R |  | 51 | -61 | 34 | 6,31 | <,001 |
|  | Supramarginal gyrus | R |  | 48 | -43 | 37 | 6,26 | <,001 |
|  |  | L |  | -48 | -46 | 37 | 7,63 | <,001 |
|  | inferior parietal | R |  | 51 | -46 | 43 | 6,04 | <,001 |
|  |  | L |  | -45 | -46 | 43 | 6,87 | <,001 |
| Occipital | Middle occipital gyrus | R | 5222 | 42 | -73 | -17 | 4,47 | <,001 |
|  |  | L |  | -48 | -67 | -17 | 5,95 | <,001 |
| Cerebellum | Declive | L |  | -24 | -73 | -29 |  | <,001 |
|  |  | R |  | 27 | -67 | -29 |  | <,001 |
|  | Inferior semi-lunar lobule | L |  | -33 | -76 | -47 | 6,45 | <,001 |
|  |  | R |  | 33 | -73 | -47 | 6,29 | <,001 |
| AD-MCI = Mild cognitive impairment due to Alzheimer’s disease; ACC = anterior cingulated cortex; FEF = frontal eye fields; PCC = posterior cingulated cortex; x, y, z = coordinates of primary peak locations in standard MNI space. For clusters with more than one peak, local maxima are listed. Cluster size in voxels. | | | | | | | | |

| Table S7. List of brain regions showing negatively correlated activity with amygdala in AD-D. | | | | | | | | |
| --- | --- | --- | --- | --- | --- | --- | --- | --- |
| **Lobe** | **Anatomy** | **Laterality** | **Cluster size** | **x** | **y** | **z** | **(z)** | **p(FWE)** |
| Frontal | Orbitofrontal | R | 57 | 24 | 44 | -17 | 5,46 | <,001 |
|  | Anterior PFC | R | 29 | 48 | 53 | -2 | 4,11 | <,001 |
|  |  | L |  | -30 | 53 | 28 | 3,24 | 0,001 |
|  | Middle frontal gyrus | R | 1512 | 42 | 35 | 34 | 3,98 | <,001 |
|  |  | L |  | -45 | 27 | 41 | 3,09 | 0,001 |
|  | Dorsolateral PFC | R |  | 39 | 38 | 43 | 5,84 | <,001 |
|  | Superior frontal gyrus | L |  | -9 | 11 | 67 | 5,62 | <,001 |
|  |  | R |  | 9 | 14 | 64 | 4,45 | <,001 |
|  | Premotor cortex | L |  | -30 | 8 | 67 | 4,39 | <,001 |
|  |  | R |  | 27 | 8 | 70 | 5,36 | <,001 |
|  | Primary motor cortex | R | 115 | 3 | -31 | 76 | 4,91 | <,001 |
|  | Caudate head | L | 927 | -12 | 11 | 1 | 5,46 | <,001 |
|  | ACC | R |  | 12 | 23 | -5 | 5,73 | <,001 |
| Parietal | Somatosensoryassociat. | L | 115 | -12 | -58 | 61 | 3,66 | <,001 |
|  | inferior parietal lobule | L | 245 | -42 | -55 | 37 | 4,85 | 4,85 |
|  |  | R |  | 45 | -55 | 40 | 4,98 | <,001 |
|  | Supramarginal gyrus | R | 510 | 48 | -52 | 46 | 5,81 | <,001 |
|  |  | L |  | -48 | -52 | 40 | 4,69 | <,001 |
|  | PCC | R | 33 | 9 | -31 | 40 | 3,78 | 0,001 |
| Temporal | Mid temporal gyrus | L |  | -61 | -52 | -7 | 4,94 | <,001 |
|  |  | R |  | 57 | -55 | -5 | 3,54 | 0,001 |
| Occipital | Mid occipital | L |  | -45 | -70 | -17 | 4,21 | <,001 |
| Cerebellum | Declive | L | 4746 | -24 | -73 | -29 | <8 | <,001 |
|  |  | R |  | 30 | -73 | -38 | 7,17 | <,001 |
|  | Inferior semi-lunar lobule | L |  | -33 | -76 | -50 | 6,72 | <,001 |
|  |  | R |  | 33 | -76 | -44 | 6,62 | <,001 |
| AD-D = Alzheimer’s disease dementia; PFC = prefrontal cortex; ACC = anterior cingulated cortex; PCC = posterior cingulated cortex; x, y, z = coordinates of primary peak locations in standard MNI space. For clusters with more than one peak, local maxima are listed. Cluster size in voxels. | | | | | | | | |

| Table S8. List of brain regions showing significantly reduced positive correlation with amygdala in AD-MCI as compared to healthy controls. | | | | | | | | |
| --- | --- | --- | --- | --- | --- | --- | --- | --- |
| **Lobe** | **Anatomy** | **Laterality** | **Cluster size** | **x** | **y** | **z** | **(z)** | **p(FWE)** |
| Frontal | Primary motor cortex | R | 32 | 45 | -16 | 49 | 3,02 | 0,001 |
|  | ACC |  | 20 | 0 | 47 | 7 | 3,89 | <,001 |
| Insula | Insula | R | 22 | 39 | -1 | 10 | 3,6 | <,001 |
|  |  | L | 88 | -39 | -4 | 7 | 3,89 | 0,001 |
| Temporal | Superior temporal gyrus | L |  | -39 | -34 | 16 | 3,92 | <,001 |
|  | Middle temporal gyrus | L | 28 | -54 | -10 | -17 | 3,89 | 0,001 |
|  | Parahippocampal cortex, HP | L | 751 | -24 | -43 | -11 | 5,9 | <,001 |
|  |  | R | 375 | 27 | -10 | -17 | 4,24 | <,001 |
|  | Inferior temporal gyrus | R |  | 33 | -34 | -20 | 5,1 | <,001 |
| Parietal | Angular gyrus | R |  | 51 | -76 | 25 | 4,18 | <,001 |
| Occipital | Superior occ. g. | R | 443 | 33 | -85 | 19 | 4,56 | <,001 |
|  | Cuneus | R |  | 12 | -85 | 46 | 4,06 | <,001 |
|  | Superior occipital gyrus | L | 751 | -39 | -70 | 7 | 4,49 | <,001 |
|  | Middle occipital gyrus | L |  | -42 | -76 | 4 | 4,13 | <,001 |
| AD-MCI = Mild cognitive impairment due to Alzheimer’s disease; ACC = anterior cingulated cortex; HP = hippocampus; Superior occ. G. = superior occipital gyrus; x, y, z = coordinates of primary peak locations in standard MNI space. For clusters with more than one peak, local maxima are listed. Cluster size in voxels. | | | | | | | | |

| Table S9. List of brain regions showing significantly reduced positive correlation with amygdala in AD-D as compared to healthy controls. | | | | | | | | |
| --- | --- | --- | --- | --- | --- | --- | --- | --- |
| **Lobe** | **Anatomy** | **Laterality** | **Cluster size** | **x** | **y** | **z** | **(z)** | **p(FWE)** |
| Frontal | ACC |  | 73 | 0 | 44 | 4 | 4,49 | <,001 |
|  | Precentral gyrus | R | 20 | 27 | -16 | 76 | 3,89 | <,001 |
|  |  | L | 282 | -27 | -19 | 76 | 4,36 | <,001 |
|  | Primary somatosensory | L |  | -42 | -16 | 67 | 3,82 | <,001 |
|  |  | R |  | 45 | -22 | 64 | 3,51 | <,001 |
|  | Premotorc., suplementary motor | R | 93 | 9 | -28 | 58 | 3,31 | <,001 |
| Insula | Insula | L | 52 | -39 | -4 | 10 | 4,03 | <,001 |
| Temporal | Parahippocampal gyrus | L | 575 | -21 | -19 | -17 | 5,62 | <,001 |
|  | Amygdala | L |  | -21 | -7 | -20 | 4,74 | <,001 |
|  | Superior temporal gyrus | L |  | -51 | 8 | -11 | 3,97 | <,001 |
|  | Parahippocampal gyrus | R | 1289 | 24 | 5 | -20 | 5,59 | <,001 |
|  | Temporopolar area | R |  | 48 | 17 | -17 | 5,21 | <,001 |
|  | Fusiform gyrus | R |  | 30 | -37 | -17 | 4,69 | <,001 |
|  | Amygdala | R |  | 24 | -7 | -14 | 3,81 | <,001 |
|  | Auditory cortex | L | 38 | -39 | -34 | 16 | 4,43 | <,001 |
| Parietal | Angular gyrus | R | 274 | 57 | -73 | 7 | 4,11 | <,001 |
| Occipital | Superior occipital gyrus | R |  | 39 | -88 | 22 | 3,84 | <,001 |
|  | Associative visual cortex | R |  | 12 | -94 | 31 | 3,82 | <,001 |
|  |  | L | 113 | -24 | -91 | 28 | 3,08 | 0,001 |
|  | Cuneus | L |  | -27 | -91 | 25 | 3,42 | <,001 |
| ACC = anterior cingulated cortex; c .= cortex; x, y, z = coordinates of primary peak locations in standard MNI space. For clusters with more than one peak, local maxima are listed. Cluster size in voxels. | | | | | | | | |

| Table S10. List of brain regions showing significantly reduced positive correlation with amygdala in AD-D as compared to AD-MCI. | | | | | | | | |
| --- | --- | --- | --- | --- | --- | --- | --- | --- |
| **Lobe** | **Anatomy** | **Laterality** | **Cluster size** | **x** | **y** | **z** | **(z)** | **p(FWE)** |
| Frontal | ACC |  | 26 | 0 | 35 | -2 | 4,04 | <.001 |
|  | Inferior frontal gyrus | R | 1216 | 21 | 8 | -10 | 6,38 | <,001 |
| Temporal | Amygdala | R |  | 21 | -2 | -23 | 5,48 | <.001 |
|  |  | L |  | -24 | -1 | -20 | 5,12 | <,001 |
|  | Temporopolar area | R |  | 48 | 17 | -20 | 4,96 | <,001 |
|  | Parahippocampal gyrus | L |  | -12 | 2 | -20 | 4,03 | <,001 |
| Insula | Insula | R |  | 42 | 5 | -14 | 4,05 | <,001 |
| AD-D = Alzheimer’s disease dementia; AD-MCI = Mild cognitive impairment due to Alzheimer’s disease; ACC = anterior cingulated cortex; x, y, z = coordinates of primary peak locations in standard MNI space. For clusters with more than one peak, local maxima are listed. Cluster size in voxels. | | | | | | | | |

| Table S11. List of brain regions showing significantly reduced negative correlation with amygdala in AD-MCI as compared to healthy controls. | | | | | | | | |
| --- | --- | --- | --- | --- | --- | --- | --- | --- |
| **Lobe** | **Anatomy** | **Laterality** | **Cluster size** | **x** | **y** | **z** | **(z)** | **p(FWE)** |
| Frontal | Anterior prefrontal | R | 71 | 45 | 56 | 4 | 4,11 | <,001 |
|  | DLPFC | R | 103 | 51 | 41 | 22 | 4,81 | <,001 |
|  | Middle frontal gyrus | R |  | 30 | 47 | 16 | 3,67 | <,001 |
|  | Premotorcortex (FEF) | L | 72 | -6 | 29 | 52 | 3,63 | <,001 |
|  | Suplementary motor area | L | 119 | -48 | 11 | 52 | 4,98 | <,001 |
|  | Middle frontal gyrus | L |  | -51 | 14 | 43 | 4,33 | <,001 |
|  | Superior frontal | R | 62 | 30 | 11 | 64 | 3,67 | <,001 |
| Parietal | Inferior parietal | L | 135 | -48 | -61 | 52 | 4,33 | <,001 |
|  | Supramarginal gyrus | L |  | -48 | -55 | 58 | 3,96 | <,001 |
|  | Superior parietal | L |  | -36 | -64 | 58 | 3,94 | <,001 |
|  | Supramarginal gyrus | R | 116 | 51 | -46 | 58 | 4,18 | <,001 |
|  | Inferior parietal | R |  | 57 | -40 | 49 | 4,15 | <,001 |
| Occipital | Lateral occipital gyrus | R | 93 | 6 | -100 | -11 | 4,11 | <,001 |
|  | Fusiform gyrus | R |  | 24 | -94 | -20 | 3,91 | <,001 |
| Cerebellum | Inferior semi-lunar lobule | R | 43 | 42 | -64 | -47 | 3,67 | <,001 |
| AD-MCI = Mild cognitive impairment due to Alzheimer’s disease; DLPFC = dorsolateral prefrontal cortex; FEE = frontal eye fields; x,y, z = coordinates of primary peak locations in standard MNI space. For clusters with more than one peak, local maxima are listed. Cluster size in voxels. | | | | | | | | |

| Table S12. List of brain regions showing significantly reduced negativecorrelation with amygdala in AD-D as compared to healthy controls. | | | | | | | | |
| --- | --- | --- | --- | --- | --- | --- | --- | --- |
| **Lobe** | **Anatomy** | **Laterality** | **Cluster size** | **x** | **y** | **z** | **(z)** | **p(FWE)** |
| Frontal | Middle frontal gyrus | L | 1368 | -30 | 59 | 13 | 5,47 | <,001 |
|  | Premotor cortex | L |  | -42 | 11 | 55 | 5,12 | <,001 |
|  | Superior frontal gyrus | L |  | -18 | 17 | 55 | 3,73 | <,001 |
|  | Anterior PFC | R | 383 | 42 | 59 | 7 | 4,88 | <,001 |
|  | Middle frontal gyrus | R |  | 39 | 56 | 22 | 4,41 | <,001 |
|  | Superior frontal gyrus | R | 312 | 36 | 23 | 46 | 4,35 | <,001 |
|  |  | L |  | 30 | 14 | 64 | 3,75 | <,001 |
|  | Inferior frontal gyrus | L | 20 | -54 | 17 | 10 | 3,48 | <,001 |
|  | Medial frontal gyrus | L | 39 | -3 | 41 | 31 | 3,2 | <,001 |
| Temporal | Mid temporal | L | 101 | -69 | -37 | -8 | 4,37 | <,001 |
| Parietal | Inferior parietal | L | 664 | -48 | -61 | 52 | 6,04 | <,001 |
|  | Precuneus | L |  | -15 | -58 | 34 | 3,98 | <,001 |
|  | Angulargyrus | L |  | -51 | -61 | 37 | 4,2 | <,001 |
|  | Supramarginal gyrus | L |  | -51 | -61 | 43 | 4,91 | <,001 |
|  | Inferior parietal | R | 335 | 54 | -40 | 40 | 3,93 | <,001 |
|  | Supramarginal gyrus | R |  | 54 | -61 | 40 | 3,84 | <,001 |
|  | PCC |  | 77 | 0 | -25 | 31 | 3,45 | <,001 |
|  | Precuneus | R | 33 | 12 | -64 | 34 | 3,16 | <,001 |
| Occipital | Lateral occipital | R | 83 | 6 | -100 | -11 | 4,65 | <,001 |
|  | Fusiform gyrus | R |  | 21 | -94 | -20 | 3,56 | <,001 |
| Cerebellum | Declive | R | 288 | 15 | -79 | -29 | 4,7 | <,001 |
|  | Cerebelar tonsil | R |  | 42 | -64 | -44 | 4,63 | <.001 |
| AD-D = Alzheimer’s disease dementia; PCC = posteriorcingulated cortex; x, y, z = coordinates of primary peak locations instand ard MNI space. For clusters with more than one peak, local maxima are listed. Cluster size in voxels. | | | | | | | | |

| Table S13. List of brain regions showing significantly reduced negative correlation with amygdala in AD-D as compared to AD-MCI. | | | | | | | | |
| --- | --- | --- | --- | --- | --- | --- | --- | --- |
| **Lobe** | **Anatomy** | **Laterality** | **Cluster size** | **x** | **y** | **z** | **(z)** | **p(FWE)** |
| Frontal | Anterior prefrontal cortex | R | 36 | 24 | 59 | 1 | 3,67 | <,001 |
|  | Superior medial frontal | R | 69 | 12 | 68 | 16 | 4,98 | <,001 |
|  | Anterior prefrontal cortex | L | 378 | -30 | 62 | 16 | 4,9 | <,001 |
|  | Middle frontal gyrus | L |  | -30 | 53 | 10 | 4,24 | <,001 |
|  | Superior frontal gyrus | L | 506 | -24 | 17 | 55 | 4,83 | <,001 |
|  | Superior frontal (FEF) | R | 79 | 27 | 26 | 49 | 3,79 | <,001 |
|  | DLPFC | R | 23 | 42 | 26 | 37 | 3,54 | <,001 |
|  | ACC |  | 132 | 0 | 29 | 37 | 3,86 | <,001 |
| Temporal | Middle temporal gyrus | L | 22 | -60 | -22 | -11 | 3,27 | <,001 |
| Parietal | PCC | L | 1360 | -3 | -43 | 31 | 3,58 | <,001 |
|  | Precuneus | L |  | -3 | -73 | 43 | 5,18 | <,001 |
|  | Angular gyrus | L |  | -45 | -70 | 37 | 4,89 | <,001 |
|  | Somatosensory association c. | L |  | -6 | -67 | 31 | 4,76 | <,001 |
|  | Angular gyrus | R | 96 | 51 | -70 | 37 | 3,95 | <,001 |
| AD-D = Alzheimer’s disease dementia; AD-MCI = Mild cognitive impairment due to Alzheimer’s disease; FEF = frontal eye fields; DLPFC = dorsolateral prefrontal cortex; ACC = anterior cingulated cortex; PCC = posterior cingulated cortex; c .= cortex; x, y, z = coordinates of primary peak locations in standard MNI space. For clusters with more than one peak, local maxima are listed.Cluster size in voxels. | | | | | | | | |

## Figure and Table Guidelines

- General Style Guidelines for Figures

The maximum number of figures and tables for all article types are shown in the [Summary Table](http://journal.frontiersin.org/journal/aging-neuroscience#SummaryTable). Frontiers requires figures to be submitted individually, in the same order as they are referred to in the manuscript, the figures will then be automatically embedded at the end of the submitted manuscript. Kindly ensure that each table and figure is mentioned in the text and in numerical order.

For graphs, there must be a self-explanatory label (including units) along each axis. For figures with more than one panel, panels should be clearly indicated using labels (A), (B), (C), (D), etc. However, do not embed the part labels over any part of the image, these labels will be added during typesetting according to Frontiers journal style. Please note that figures which are not according to the guidelines will cause substantial delay during the production process.

Permission must be obtained for use of copyrighted material from other sources (including re-published/adapted/modified/partial figures and images from the internet). It is the responsibility of the authors to acquire the licenses, to follow any citation instructions requested by third-party rights holders, and cover any supplementary charges.

Frontiers takes concerns regarding image manipulation seriously. We request that no individual features within an image are modified (eg. enhanced, obscured, moved, removed or added). Where images are grouped together, for example, parts of gels are lined up, this must be clearly explained in the figure or in the figure text. Any change in brightness, contrast or color balance must be applied to every pixel in the image and the changes should not alter the information illustrated in the figure. Any concerns raised will be investigated and the authors will be asked to provide the original images.

- General Style Guidelines for Tables

Tables should be inserted at the end of the manuscript. If you use a word processor, build your table in word. If you use a LaTeX processor, build your table in LaTeX. An empty line should be left before and after the table.

Please note that large tables covering several pages cannot be included in the final PDF for formatting reasons. These tables will be published as supplementary material on the online article abstract page at the time of acceptance. The author will notified during the typesetting of the final article if this is the case. A link in the final PDF will direct to the online material.

- Figure and Table Legends

Figure and table legends are required to have the same font as the main text (12 point normal Times New Roman, single spaced). Legends should be preceded by the appropriate label, for example "Figure 1" or "Table 4". Figure legends should be placed at the end of the manuscript (for supplementary images you must include the caption with the figure, uploaded as a separate file). Table legends must be placed immediately before the table. Please use only a single paragraph for the legend. Figure panels are referred to by bold capital letters in brackets: (A), (B), (C), (D), etc.

- Image Size

Figure images should be prepared with the PDF layout in mind, individual figures should not be longer than one page and with a width that corresponds to 1 column or 2 columns.

- - **All articles are prepared using the 2 column layout:** 2 column articles can contain images 85 mm or 180 mm wide.
- Format

The following formats are accepted:
TIFF (.tif) TIFF files should be saved using LZW compression or any other non-lossy compression method.
JPEG (.jpg)
EPS (.eps) EPS files can be uploaded upon acceptance

- Color Image Mode

Images must be submitted in the color mode RGB.

- Resolution Requirements

All images must be uploaded separately in the submission procedure and have a resolution of **300 dpi at final size**. Check the resolution of your figure by enlarging it to 150%. If the resolution is too low, the image will appear blurry, jagged or have a stair-stepped effect.

Please note saving a figure directly as an image file (JPEG, TIF) can greatly affect the resolution of your image. To avoid this, one option is to export the file as PDF, then convert into TIFF or EPS using a graphics software. EPS files can be uploaded upon acceptance.
